# Observations on the Effects of the Digitalis Purpurea, in the Cure of Phthisis Pulmonalis

**Published:** 1801-03

**Authors:** J. Magennis

**Affiliations:** Royal Hospital


Medical and Phyfical Journal.

VOL. V.] MARCH, 1801. [NO. XXV.

### *Obfervations on the Effects of the Digitalis Purpurea, in the Cure of Phthifis Pulmonalis.*

To the EDITORS of the MEDICAL and PHYSICAL JOURNAL.

Gentlemen,

THE public attention has been fixed with a confiderable degree of anxiety for the laft two years, on the various contradictory accounts, publifhed at different times, refpecting the effects of the Digitalis as a fuccefsful remedy in the cure of pulmonary confumption. Like every new and valuable difcovery that has been made and applied to the removal of one or more of the afflicting catalogue of difeafes to which the human frame is fubject, the Fox-glove has experienced the moft unbounded praifes, and the keeneft cenfure. The advocates for the ufe of this medicine, as is ufual with all new difcoveries, have extolled its virtues probably beyond the juft bounds of truth, and what will not be found warranted by a more general and extenfive experience. On the other hand, its enemies have not only denied its poffeffing any antiphthifical powers whatever, but have abfolutely condemned it as a dangerous and deleterious drug, which ought to be altogether excluded from medical practice, and ranked only in the clafs of the moft deadly vegetable poifons. The truth is moft commonly found to lie between thefe extremes; and in no inftance, perhaps, is this general rule more applicable than in the prefent.

Every profeffional man, who fets a juft value on his own character, who feels interefted in the fate of fuffering humanity, and in the prefervation of thofe more immediately committed to his care, muft perceive the neceffity of a cool and temperate inveftigation of a fubject, which involves in its confequences the fafety or deftruction of thoufands of his fellow creatures. Few, I hope, will differ with me in this opinion; and yet, if it were to be decided by a reference to the many publications which annually iſſue from the preſs, I am very apprehenſive the queſtion would be determined againſt me. If we enquire into the cauſes of theſe inconſiſtencies and contrarieties, they will be found in the various paſſions which but too commonly agitate the human breaſt; envy, jealouſy, claſhing of intereſts, in a ſpirit of oppoſition and rivalry.

If the publications before alluded to were the ſole rule to regulate our judgments by, it would appear from the aſperity of language employed, that in medical diſquiſitions, as in thoſe of politics and religion, the inveſtigation and diſcovery of truth do not form the baſis of diſcuſſion, or the great object contended for; but an anxious deſire on the part of the diſputants of convicting each other of mis-ſtatement and error, with an ardour and animoſity which always render the parties leſs reſpectable in the eyes of every diſpaſſionate and enlightened obſerver. It is true, that from the circumſcribed and limited nature of the human intellect, men even of the moſt enlarged ideas are frequently liable to error, and they would be ſtill more ſo, were they under no apprehenſions of having their opinions canvaſſed, their ſentiments controverted, and their works ſubjected to the laws of juſt criticiſm. When opinions are examined with calmneſs and impartiality, but with a bold and temperate freedom, the boundaries of ſcience are ſure to be extended and enlarged, and truth, as far as it is attainable by diſcuſſion, will become the certain reſult.

Two gentlemen, to whom the public and the profeſſion are certainly under obligations for the light they have thrown on the nature and properties of Digitalis, have, to the manifeſt injury of the cauſe they were defending, diſputed with intemperance, to ſay nothing elſe of it, on this intereſting ſubject: But I am happy, however, to ſee the contention likely to terminate by an effort of forbearance on the one part, which does the author honor; and it would undoubtedly be to the advantage of ſcience and urbanity, if the learned Editors of this Journal oppoſed as much as poſſible, diſputations carried on with animoſity, where paſſion uſurps the ſeat of reaſon, and prejudice that of truth.

It is the duty of every medical man, to exert his utmoſt abilities in endeavours to oppoſe barriers to the waſteful voids occaſioned ſo frequently in families and ſociety by the fatal effects of pulmonary conſumption; and he who devotes his leiſure and talents ſucceſsfully, in purſuit of means to check the career of this deſolating ſcourge, muſt deſerve well of his country and humanity.

During the time I had the charge and ſuperintendance of the Hoſpitals at Norman Croſs, I had occaſion to try the effects of the the Digitalis on consumptive patients; and from the fortunate termination of Stroed's case, related at large in No. XII. page 128 of this Journal, I was extremely desirous of employing it still more extensively;—opportunities were not long wanting. On the 22d of Dec. 1799, the French government, in violation of its former engagements, thought proper to decline victualling the prisoners any longer in England; in consequence of this dishonourable step, I had orders from the Commissioners of sick and wounded Seamen, to take charge of the French sick as heretofore. I found in the wards, recently under the management of the Republican surgeons, eight patients, who had been in the hospital from four to seven months and upwards, all deeply affected by Phthisis. Six were in its last and most confirmed stage, and two in the second, supposing three stages an accurate division of the disease: Four out of the six were in so deplorable a state, that under any system of treatment their cases appeared quite desperate and hopeless. Thus circumstanced, they began the use of the Fox-glove in the form of tincture, which was gradually exhibited, and continued for three weeks; at the end of this time they were so much amended, that I began to entertain the pleasing and consoling hope, my endeavours would ultimately be crowned with success. The expectoration diminished to nearly one half; the cough considerably abated; the nightly perspirations, except in one, wholly disappeared; the pulse, in all of them, had gradually fallen from 100 and 110, to between 50 and 65; and the harrassing pains about the thorax and its vicinity were very generally relieved: But alas! these encouraging appearances were of short duration, and continued only a few days longer. The weather, which had been mild, and extremely favourable for the season, suddenly changed to cold sleet and frost, with a north-east wind, which, from the exposed site of the prison, its proximity to a large lake of water, and a marshy country lying east of it, blows here uncommonly keen. The consequence of these alterations was, that fresh excitement took place; the cough, expectoration, and all the other symptoms became highly aggravated; and after a long and severe struggle, five out of the eight fell a sacrifice to the malignity of the disease; two permanently recovered, and one was very much relieved; but being sent to France, I have had no opportunity of learning the issue of the contest. Justice obliges me to acknowledge, that the two who completely recovered were less affected than the others, though their expectoration was evidently purulent. From the extraordinary amendment which occurred in the commencement of their treatment, I am strongly inclined to believe, that, had the weather preserved the same

 degree degree of temperature one month longer, three at leaft out of the five, if not the whole, would have been probably reftored to health.

Shortly after this period I was removed to the Royal Hofpital at Plymouth, a fituation which affords to medical induftry a wider range, and more extenfive field for obfervation, in the profecution of a fubject fo interefting to humanity; a fituation in which the powers, properties, and general effects of the Fox-glove on the animal œconomy may be afcertained to a degree of exactnefs and precifion, nearly approaching to mathematical certainty, if practical experience in variety and number of cafes, in every ftage of the difeafe, is to form the criterion on which we are to ground a decifive opinion, taking at the fame time into confideration, various adventitious circumftances, as climate, or temperature of the atmofphere, former habits, and the peculiar idiofyncrafy of the different objects intended to be fubjected to its powerful influence.

The number of patients fent to the Naval Hofpital in the laft ten months, and the mortality caufed by this difeafe among the feamen and marines of his Majefty's fleets, are truly alarming; a great portion of the men admitted during the above period actually laboured under Phthifis, either in its incipient or moft confirmed ftage, but principally of the latter defcription.

Among the caufes that have contributed to increafe the number affected with this confuming malady may be mentioned as a principal, the continual and fevere duty in which the channel fleet has been employed for many months paft, being feldom more than a few days in port, and that only when abfolute neceffity compelled it, to victual and water.

Of the prodigious numbers received from the fhips of war and marine barracks, labouring under Phthifis Pulmonalis in its incipient or confirmed ftage, I have here felected feventy-two patients who were fubjected to the powerful influence of the Digitalis; but as the limits of the Medical and Phyfical Journal cannot poffibly admit of a detailed ftatement of fuch a variety of unhappy cafes, I fhall give only the hiftory of three or four of the moft marked and decifive, in which the operation and good effects of the medicine were confpicuous; the remaining number, as well as thofe narrated more at large, I infert in a Table, with the general refults, as the moft concife mode of exhibiting fuch an unwieldy body of evidence.

William White, aged about 28, a feaman belonging to his Majefty's fhip Gibraltar, was admitted on the 12th of Auguft, 1800, in the laft and moft confirmed ftage of phthifis pulmonalis. He had been ill for feveral months with cough continually increafing in feverity, and which was now inceffant; pain pain in both fides of the thorax, efpecially in the left, running along the cartilaginous extremities of the ribs; expectoration profufe of greenifh pus, extremely fœtid, now and then ftreaked with blood; heavy perfpirations, with febrile exacerbations night and morning. He particularly complained of a throbbing pain feated between the feventh and eighth ribs; and the cutis covering this part, externally, was difcoloured in a very curious manner, about the circumference of a crown piece: The refpiration oppreffed and laborious; he could only lie on his back; and the pulfe beat 108 ftrokes in a minute.

Under this accumulated ftate of difeafe and wretchednefs, no very fanguine hopes could be entertained of his recovery; in fhort, I confidered him as one out of the great many unfortunate objects, fent to the Hofpital to die in a few days. May I not afk here with propriety, why this man had been kept fo long on board fhip in fuch a deplorable ftate? He began the Digitalis, however, this day, in dofes of feven drops every four hours, in a fperm. cæti mixture with tinct. opii camp. On the 13th, after a fevere fit of coughing, he brought up half a pint of very offenfive matter. Repe. Tinct. Digital. gt. L. 15th, Tinct. gt. LX. 16th, LXX. 17th and 18th, No alteration in his condition; the tincture increafed by ten drops daily. 19th, Tinct. gt. C. flight naufea; expectoration copious; cough fevere; allowed four or five glaffes of wine daily; pulfe 90. From the 20th to the 24th, the medicine regularly augmented in the proportion before mentioned. 25th, The expectoration not fo abundant, with lefs fœtor; tinct. gt. CL. pulfe 78. 26th, Tinct. gt. CLX. 27th, Tinct. gt. CLXX. 28th, Tinct. gt. CLXXX. 29th, Naufea, with vomiting, occurred yefterday; diminifhed the tinct. to gt. CXXX. pulfe 48, and irregular. 30th, Naufea almoft removed; pulfe 48, more fteady. 31ft, Pain and oppreffion about the præcordia; App. empl. canthar. fcrob. cord. Naufea and ficknefs entirely gone; expectoration vifibly diminifhing; pulfe 52; tinct. gt. CXL. Sept. 1ft, Tinct. gt. CL. The 2d, 3d, 4th, and 5th, the tinct. regularly increafed. 6th, Tinct. gt. CLXXX. Naufea, vertigo, with an intermittent pulfe at 46. 7th, Naufea and dizzinefs continue, but the cough greatly abated; the expectoration reduced to half the original quantity; the greenifh hue removed; the fœtor gone; fcarcely any perfpirations for the laft three nights. 8th, Tinct. gt. CC. 9th, Naufea in the morning; pulfe irregular, and intermitted for feveral days. 10th, Tinct. gt. CCXX. 11th, 12th, and 13, Naufea with flight vomitings, prevented an augmentation of the tincture during the laft three days; pulfe from 44 to 50, irregular; expectoration aftonifhingly diminifhed, and every other fymptom much abated.

14th,

14th, Naufea and vertigo gone; Tinct. gt. ccxxx. 16th, Scarcely any cough now remaining. 17th, Tinct. gt. ccxxx. flight naufea; pulfe 48. 18th, Tinct. gt. ccxl. 19th, Tinct. ccl. expectoration reduced to a table fpoonful. 22d, Hardly a veftige of difeafe remained; every part of the thorax completely freed from pain; even his ftrength was much amended. As the difeafe removed, he could bear larger dofes of the medicine, which was ftill continued. 25th, With a pulfe at 56, he was as free from complaint as at any one period of his life, bodily ftrength alone excepted: The Digitalis ftill perfevered in and augmented. On the 29th of Sept. he was difcharged the hofpital, perfectly reftored to health. A few days after his reception, he was invalided as a hectic patient, there being little hope at the time of his ever leaving the ward in a living ftate. Of this, the man was himfelf fo fenfible, that he has fince written a letter, acknowledging his obligations in the ftrongeft terms of gratitude.

James Smith, aged 26, a feaman belonging to his Majefty's fhip Ville de Paris, admitted on the 6th of October laft. Has been ill a long time with phthifis; he was affected with every fymptom which is the ufual concomitant of the difeafe in its laft and moft aggravated ftage; conftant and deep feated pain in both fides, but more particularly in the left; expectoration profufe, and rankly purulent, emitting a moft difagreeable fœtor; regular attacks of febrile exacerbations every evening; profufe colliquative fweats; formerly fubject to frequent hæmorrhages from the lungs, but nothing of that kind had occurred lately: He was reduced to the loweft ftate of debility, although twelve months before of Herculean powers, as one of his mefs-mates informed me. The moment I faw this man, I pronounced it a loft cafe: Notwithftanding thefe unfavourable appearances, he began the Tinct. Digital. in fmall dofes, which were gradually increafed, and fyftematically perfevered in till the 21ft of Nov. on which day he was difcharged, cured. The expectoration, originally a pint and a half, was reduced to about a table fpoonful or lefs, and wholly free from purulence; the nightly perfpirations had ceafed upwards of twelve days; the thorax was completely freed from pain; and the cough had for fome days totally difappeared, except for a few minutes after his firft getting out of bed in the morning. I wifhed much to put him on a courfe of chalybeate tonics before his difcharge; but having been previoufly invalided, he became extremely anxious to fee his friends in Ireland, and ftill more fo, fearing that an alteration in the weather might give him frefh cold in travelling, fhould the froft fet in before his departure. He was confined to his bed near three weeks whilft under the influence influence of the medicine, in ſuch an extreme ſtate of inſenſibility all the time, that at firſt he could only get out to the night-chair with the aſſiſtance of a nurſe. The pulſe gradually fell from 110 to 60, but never lower; and for a long time the tincture could not be puſhed beyond 100 drops in the day; I was twice under the neceſſity of reducing the daily quantity to 50 drops: But as the cure advanced and his ſtrength increaſed, he could bear larger doſes; for ten days previous to his diſmiſſal, he took to the amount of 160 drops a day; beyond that, the medicine diſordered the head and ſtomach.

Mr. William Campbell, of his Majeſty's ſhip Barfleur, 23 years of age, was admitted on the 27th July, 1800, labouring under Phthiſis. He was greatly emaciated, expectorated confirmed pus, a fixed pain of the ſide, a deep hollow cough, febrile exacerbations, profuſe nightly perſpirations, a pearly whiteneſs of the eyes, great thirſt, oppreſſed and difficult reſpiration, head-ach, and a pulſe above 100 in a minute. On this day he began the uſe of the Digitalis, which was by degrees increaſed till the daily quantity amounted to 160 drops; this was the utmoſt extent to which the tincture could be puſhed, without diſordering the head and ſtomach. The pulſe gradually ſunk, the cough and expectoration at length wholly diſappeared, every other hectic ſymptom by degrees vaniſhed, and on the 7th of September he was diſcharged, completely reſtored to health.

James Herritage, ſeaman, 29 years of age, was admitted for the ſecond time into the hoſpital on the 14th Auguſt, 1800. He had long been ſubject to ſevere pain of the breaſt, inceſſant cough, purulent expectoration, ſometimes mixed with blood, much emaciation, loſs of appetite, febrile rigors attended with colliquative and profuſe ſweats at night, with a pulſe at 108. The Digitalis was, as uſual, exhibited in ſmall doſes, which were ſyſtematically increaſed by ten drops a day, until towards the termination of his cure he could bear the aſtoniſhing doſe of 100 drops three times a day. I repeatedly and perſonally exhibited the medicine myſelf. He was ſurveyed, and would have been invalided; but at my requeſt was permitted to remain, as I then judged him faſt advancing towards a perfect cure, in which I happily ſucceeded. The pulſe gradually ſunk to 60 pulſations in a minute, but never fell lower, notwithſtanding the enormous doſes which he took of the medicine. He was diſcharged to duty on the 29th September, forty-three days after his reception.

James Wallace, a ſeaman, was received into the hoſpital on the 14th September laſt. He had at this time been two years and upwards ſubject to cough; he now complained of a deep

ſeated feated pain in the right fide, and about the fcrob. cord. expectorated a thin fanious and purulent matter, emitting a moft intolerable ftench; the fœtor of his breath was fo extremely offenfive, that I was conftantly obliged to turn my head afide when examining into the ftate of his pulfe, and the nurfes were often affected by naufea and ficknefs when in the act of affifting him with drink, &c. a laborious and oppreffed refpiration; but he had neither rigors nor night fweats, except in a very trifling degree. The pulfe fell by regular gradation from 104 to 46, and continued in this extraordinary ftate of depreffion for five weeks, never afcending higher than 54 all the time: he could never go beyond 100 drops of the tincture in 24 hours, and yet this quantity was fufficient to bring down and keep the pulfe at the extreme reduction above-mentioned.

He was difcharged cured on the 28th of November, 1800. As I had not the moft diftant hope of this man's recovery, confidering him in a cadaverous or femi-putrefcent ftate at the commencement, he was fubmitted to furvey, and accordingly invalided.

In thefe great variety of cafes, the medicine was frequently given under my own immediate infpection, or that of my affiftants, efpecially when the dofes amounted to what is deemed very large, in order to be certain that we were not deceived by the patients. Many were in the habit of taking from 150 to 300 drops in the day, firft commencing with 20, 30, 40, or 50 drops, regulating the quantity according to the apparent ftrength and vigour of the patient; and increafing the number daily by ten drops, till the ftomach began to fhow flight fymptoms of anorexia, or the patient complained of dizzinefs and imperfect vifion, accompanied with a confiderable reduction of the pulfe: Whenever one or more of thefe fymptoms occur, the dofes are immediately reduced, either one half or the quantity originally commenced with; but if the head and ftomach fhould ftill be unable to bear thefe reduced dofes from the irritation previoufly induced, a circumftance, however, to be fedulouſly guarded againft, the medicine is then wholly omitted for one, two, or three days, after which it is again exhibited as at the firft onfet.

The vehicle in which I ufually adminifter this medicine is, a mixture of Sper. Ceti. Tinct. Op. Cam. et Ox. Scil. from two to three table fpoons full, to be taken every four or fix hours; fo that the quantity with which the patient generally commences, is from feven to ten drops at a dofe. It is fometimes exhibited in Decoct. Cinch. c. Elix. Vit. et Tinct. Cinch. and the formula varied according to the particular urgency of fymptoms. The patient is ufually permitted to take a few glaffes of wine daily, daily, and ſometimes to the quantity of a pint, when coll quative ſweats and great debility render it neceſſary.

By theſe means, and by theſe precautions, I have been able to exhibit it to the great extent already ſpecified.

In comparing theſe quantities with the doſes adminiſtered by ſeveral of your correſpondents in private practice, I have been much ſtruck with the great and material difference between us, as thoſe gentlemen were ſeldom able to go beyond 90 or 110 drops; and that in few inſtances. To what can we impute the cauſe of theſe contrarieties of reſults? Either it muſt ariſe from the inferior powers of the plant of which the tincture is here made; from the imagination of a certain deſcription of patients; from the few opportunities occurring in private practice of trying the Digitalis on a variety of conſtitutions; or from a combination of ſome or all theſe circumſtances. With reſpect to the firſt, Mr. Hammick, the diſpenſer of the hoſpital, informs me, that the Fox-glove of which the tincture is made, is commonly procured from the Hall, and the proportions are 1 oz. groſs powder, to 4 oz. of proof ſpirit. 2dly, Private patients being in ſome meaſure acquainted with the active properties of the medicine, and ſeeing it dropt with great caution, expect from it certain effects, with which the imagination is fully impreſſed. And laſtly, many conſtitutions will hardly bear the ſmalleſt doſes, without exciting very general tumult in the ſyſtem. I have now ſeveral under my care that cannot go beyond 70 drops a day, and two who are not able to bear 40 drops, or ſix at a doſe.

Mr. Fuge, firſt ſurgeon to the hoſpital, having directed his attention to this ſubject, and in order to enſure greater accuracy, was at the trouble of collecting the plant himſelf, and of making the tincture, in the proportion of four ounces of freſh gathered leaves to five of proof ſpirit: Of this tincture he had the politeneſs to ſend me eight ounces; and I found on trial, that ten drops of it were equal to nearly fifteen of what I was then in the habit of preſcribing. This circumſtance demonſtrates fully the abſolute neceſſity of a ſtandard and fixed formula from authority, to regulate general practice by; and likewiſe the neceſſity of great caution in the exhibition of this valuable medicine, before the ſtrength of the tincture is accurately aſcertained.

The effects of the Digitalis on different conſtitutions, is ſtrongly exemplified in two patients now under my care: In one, the medicine cannot be puſhed to forty drops a day, without inducing nauſea, vertigo, and very general derangement of the ſyſtem; the other, Robert Skinner, has taken one hundred drops three times a day, without producing the ſmalleſt uneaſi nefs whatever, not even intermiffion or irregularity of the pulfe, an effect that very generally follows the ufe of this medicine when given in full dofes. A confiderable reduction of the pulfe, however, took place, which the following morning was fteady and regular at 60 pulfations in a minute; the medicine being wholly omitted the following day, the pulfe rofe to feventy-fix. I have met with feveral inftances in which the Digitalis, given freely and largely, effected not the fmalleft reduction in the pulfe; and in thefe, the patients uniformly derived no advantage whatever from its ufe. I fhall briefly fubjoin two cafes to illuftrate this point; one occurred at Norman Crofs, the other at the Royal Hofpital.

Henry Velcamp, a Dutch prifoner of war, but a Frenchman by birth, was admitted into the hofpital repeatedly during the laft ten months, generally for the cure of pleuritic affections.—Some time before the 25th of October, 1799, he complained of pain in the left fide, as he had indeed often done before, fevere cough, copious expectoration though not purulent, difficult refpiration with a fomewhat hard pulfe and a conftipated ftate of the bowels. He was immediately prefcribed a faline cathartic, a large blifter to the part affected, and a mixture of Sperm. ceti. aq. am. acet et fal. nitr. This plan was perfevered in till the 30th, with little or no amendment. On this day ordered Tinct. Digit. gt. xv. bis die, in two ounces of the above mixture.—Nov. 1, Tinct. gt. xv. ter die.—2d, Tinct. gt. xx. ter die. The tincture was regularly increafed by five drops a dofe, till he took 165 drops daily. On the 9th I was quite aftonifhed that no alteration followed with refpect to the head, ftomach, and in the number of pulfations, being never fewer from the commencement than 100 to 110. Anxious to examine the condition of the blood, I directed three ounces to be taken away; but notwithftanding the rapidity of the circulation, and the apparent wirinefs of the pulfe, the blood fhewed no figns of inflammation. The tincture was continued till the 12th, on which day he took 210 drops, that is, 70 drops three times a day; but finding no alteration in the pulfe, or general ftate of the patient for the better, on the contrary he was daily getting worfe, the Digitalis was difcontinued altogether; he lived only fix days longer, notwithftanding the application of other remedies, and fell a victim to the diforder on the 18th of November. From the unufual fymptoms attending this cafe throughout, and the failure of the Digitalis in abating the force of the vafcular fyftem, I was very defirous of examining into the ftate of the vifcera. The body was accordingly opened by my head affiftant, Mr. Woodham. The liver was found much enlarged, with the appearance of an incipient incipient gangrene along its extreme edge ; the gall bladder was nearly empty, containing only a ſmall quantity of a dark viſcid bile; the lungs were full of tubercles, though few in a ſtate of ſuppuration ; no adheſions to the pleura had taken place, and all the other viſcera were perfectly ſound.

Benjamin Eve, a ſeaman, belonging to his Majeſty's ſhip Superb, a young man about 22 years of age, was admitted on the 26th September laſt, labouring under Phthiſis, his countenance ſtrongly indicating the nature of his diſeaſe. He was ſubject to nightly ſweats, attended with hectic rigors morning and evening, copious expectoration of bloody pus, and conſtant cough. He had been ſubject to frequent hæmorrhagies from the lungs for ſome months previous to his admiſſion, and although not ſo frequent now, yet they ſometimes did occur. He began the Digitalis on the 27th, which was regularly continued and gradually increaſed till the daily quantity amounted to 230 drops ; this was on the 42d day from its commencement. During all the time, it neither abated the force or reduced the frequency of the pulſe, which was never under 100, but moſt commonly at 110 to 120; nor did the diſeaſe receive the ſmalleſt apparent check in its career. He was about this time invalided and diſcharged at his own requeſt, ſoon probably to fall a victim to this horrible malady. Here is a caſe in which the Digitalis entirely failed, where it had a fair trial. I might adduce others, to prove that the effects of this medicine will be various and diverſified on different conſtitutions.

I have never perceived in any one inſtance the ſmalleſt tendency in the medicine to act on the kidneys, even when given in the largeſt doſes ; and this is the more extraordinary, as it was firſt introduced to the public notice under the character of a powerful remedy in the cure of dropſy. The Digitalis not only failed on all occaſions of having any diuretic effect; but what is ſtill more to be wondered at is, that it actually produced, and has been the cauſe of the very diſeaſe in queſtion; an effect which might naturally be ſuppoſed to ariſe from it in reaſoning theoretically on the properties of this plant, from its ſedative operation on the force and vigour of the vaſcular ſyſtem. This is a conſequence which I ſay might naturally be deduced from theory, and which will now be ſeen confirmed by the following facts. It is therefore neceſſary to point out to the inexperienced practitioner, the poſſibility of ſuch an occurrence, that in his endeavours to remove one diſeaſe, he may not ſubſtitute another equally fatal.

Two inſtances have lately occurred in the courſe of my practice at the Royal Hoſpital, which confirm the juſtneſs of the above obſervations ; and that I may not ſwell this article beyond all moderate bounds, I fubjoin one cafe only. David Johnfton, a young lad, about 17 years of age, the fon of one of the nurfes, had long complained of fevere cough, oppreffed and difficult refpiration; his expectoration was now purulent, his eyes of a pearly whitenefs, which is often a fure indication of confirmed phthifis; he was much emaciated in confequence of nightly fweats, attended with regular febrile exacerbations, and a pulfe above 100 in a minute. In fhort, this lad was fo ill that I expreffed my opinion to Mr. Peters, phyfical affiftant, that it was probably a loft cafe. The tincture was however exhibited in reduced dofes, which were gradually increafed and regularly continued till the end of September, having commenced about the clofe of the previous month; and to my very great furprife all the hectic fymptoms were by this time entirely removed; a very extraordinary and rapid cure in a cafe of phthifis fo ftrongly marked. The medicine was ftill continued, but in a few days his lower extremities began to fwell, which rapidly extended to the trunk, and prefently after he was univerfally anafarcous with evident fluctuation in the cavity of the abdomen. The Digitalis was now wholly omitted, and the patient ordered the chalybeate diuretic mixture, the good effects of which I had already fo happily experienced in the cure of dropfy; and it proved equally fuccefsful in the cafe at prefent under confideration. In fourteen days the lad was completely reftored to health, which ftill continues up to this moment.

This is a cafe, which, when confidered with attention, and duly reflected on, muft throw great light on the nature, properties, and modus operandi of the Fox-glove; it gradually and filently funk the vigour, and abated the force of the whole fyftem, but moft confpicuoufly fo that of the vafcular; the pulfe fell in a few days from 100 in a minute to under 50, and was retained in this ftate of depreffion, till every fymptom of phthifis had wholly difappeared; this being a difeafe having principally for bafis, irritation, and a preternaturally increafed velocity of the circulation, ultimately inducing particular local affection, which, by conftant attrition, exhaufts the fenforial power and wears down the conftitution. The morbid catenation of affociated fymptoms once deftroyed, or in other words, the over-plus of preternatural action in the minute and fecretory veffels of the lungs once reduced to the healthy ftandard, the fyftem ftill continuing under the powerful influence and fedative effects of the medicine, a difeafe of diminifhed excitement, the reverfe of the former, is fuper-induced; that is, dropfy became the confequence, which in its turn yielded to the ftimulus and invigorating powers of fteel combined with diuretics.

In

In maturely weighing all the facts and circumſtances here adduced, I am inclined to impute the ſalutary influence exerted by the Digitalis over Phthiſis, to its widely diffuſing property of diminiſhing morbid excitement throughout the animal œconomy; and not to any peculiar ſpecific power it poſſeſſes of promoting abſorption either general or local, which is ſuppoſed to be its modus operandi in the cure of dropſy and phthiſis.—For it appears contrary to analogical reaſoning drawn from the known laws of the animal œconomy, that a medicine which ſo forcibly retards the motion of the heart and arteries, ſhould at the ſame time increaſe that of the abſorbent ſyſtem; a conſequence that muſt neceſſarily ariſe, in order to remove fluids depoſited in the different cells and cavities of the body.

It is not then the rapid abſorption of ſecreted and aerated pus from the ſurface of diſeaſed parts, on which the cure of phthiſis depends, but on the diminution and total extinction of morbid increaſed action in the extremities of the pulmonic veſſels; thereby cutting off the ſole ſource and cauſe of ſecretion; which, when once effected, the diſeaſe ceaſes to exiſt. This opinion is ſtill farther corroborated, when it is recollected, that in all thoſe caſes wherein the tincture failed of effecting a reduction of the pulſe, no amendment followed its uſe.

To adminiſter the Digitalis under every favourable circumſtance, and to render its ſucceſs the more certain, it is of the utmoſt importance to attend carefully to the ſtate of the atmoſphere; frequent changes and ſudden tranſitions of temperature are ſeduloufly to be guarded againſt: The practitioner who loſes ſight of this conſideration, or thinks lightly of it, will be generally foiled in his efforts to cure Phthiſis Pulmonalis, even when aſſiſted by the powerful effects of the Fox-glove. A ſtate of weather which commands a range of the thermometer, lying between 55 and 65 degrees, is the beſt adapted to the ſucceſsful treatment of hectic patients. Winter is therefore the moſt unfavourable ſeaſon for thoſe labouring under phthiſis; and I am fully convinced, that many fall victims to the diſeaſe at this period of the year, that would moſt probably have recovered in ſummer. Beſide the caſes in point already adduced to ſupport the truth of this aſſertion, I had two remarkable inſtances at the Royal Hoſpital. John Wilſon, now no more, after uſing the Digitalis for five weeks, was conſiderably better; but on the approach of winter, the weather alternating frequently with heat and cold, freſh excitement took place, and proved fatal to him. The other, Jameſon, ſtill living; but who, without the immediate interpoſition of Omniſcience, muſt likewiſe die, was at one time ſo far recovered, that I conſidered his cure certain. He was admitted in the laſt ſtage of Phthiſis, and from a pint of of pure matter, the expectoration diminifhed to about two table fpoons full, and that not purulent. Change of temperature induced a relapfe, and he is now on the verge of eternity. The Tinct. Digit. had in thefe two cafes a complete trial, but failed.

I have chofen the annexed tabular form, as conveying at one luminous view the whole of the information which I have collected on this important queftion, from the opportunities afforded by my ftation at the Royal Hofpital. By this, with what has been already publifhed by other gentlemen, the public and the profeffion will be enabled to appreciate the real value, or at leaft make a much nearer approach in appreciating the real value of this extraordinary medicine. They will perceive, that although it will cure Phthifis in its moft advanced and aggravated ftage; yet, that it will fail in many inftances of a fimilar nature, and that, even when the difeafe is ftill in its incipient ftate. To promife and to expect more from this or any other medicine, in the treatment of general difeafes, will, I fear, be holding out falfe lights to deceive inexperience, to wound truth, to injure and retard fcience.

It is of great confequence that the properties of this medicine fhould be accurately defined, as the cautious and timid practitioner, and thofe whofe fphere of practice is limited to few opportunities of trying the effects of new and doubtful remedies, muft wait the decifion of this queftion with an impatience and folicitude proportioned to the weight and importance of the fubject.

Upon the whole, it will be found a valuable addition to our ftock of knowledge in Therapeutics, already under fo many obligations to this age of improvement; but too often, of unhappy innovation.

I cannot quit this fubject, without firft acknowledging many obligations to Doctor Drake, for having attracted my attention to the valuable properties of the Digitalis, in his accurate narrative of its effects on Maris and Grimes, publifhed in Doctor Beddoe's volume of Contributions.

I remain, &c.

*Royal Hofpital,* 20 *Jan.* 1801. J. MAGENNIS, M.D.

P. S. In a paper of mine on Epilepfy, page 419 of No. xxi. of your Journal, an error has crept in; inftead of irritability, it fhould run, general *inirritability*.

NAMES

| NAMES. | STAGE. | RESULT. |
|---|---|---|
| Matthew Hall, S. | Purulent. | Recovered. |
| Gregory M'Donnald, S. | Purulent. | Recovered. |
| Thomas Davis, S. | Purulent. | Recovered. |
| James Craig, S. | Purulent. | Recovered. |
| Henry Downing, S. | Purulent. | Recovered. |
| William Hill, M. | Purulent. | Recovered. |
| John Caruthers, S. | Purulent. | Recovered. |
| Jofeph Cork, M. | Purulent. | Recovered. |
| Mr. W. Campbell. | Purulent. | Recovered. |
| William Hammond, S. | Purulent. | Recovered. |
| Thomas Swift, S. | Purulent. | Recovered. |
| William White, S. | Purulent. | Recovered. |
| James Herritage, S. | Purulent. | Recovered. |
| William Thorn, M. | Purulent. | Recovered. |
| William Grin, S. | Purulent. | Recovered. |
| Robert Searle, S. | Purulent. | Recovered. |
| James Smith, S. | Purulent. | Recovered. |
| James Wallace, S. | Purulent. | Recovered. |
| William Brown, S. | Purulent. | Recovered. |
| David Johnfon. | Purulent. | Recovered. |
| Robert Skinner, S. | Purulent. | Recovered. |
| —— Kennedy, S. | Purulent. | Recovered. |
| —— Wallace, S. | Purulent. | Recovered. |
| James Karns, M. | Purulent. | Recovered. |
| Patrick Byrne, M. | Purulent. | Recovered. |
| Paul Laurence, M. | Incipient. | Recovered. |
| James Hardy, S. | Incipient. | Recovered. |
| John Cleverly, M. | Incipient. | Recovered. |
| John Wilkins, S. | Incipient. | Recovered. |
| Michael Raferty, S. | Incipient. | Recovered. |
| James Ruffel, M. | Incipient. | Reeovered. |
| Roger Roony, S. | Incipient. | Recovered. |
| Michael Anderfon, S. | Incipient. | Recovered. |
| Mr. J. Weft, furgeon's mate. | Incipient. | Recovered. |
| Cornelius Dogherty, S. | Incipient. | Recovered. |
| Samuel Beft, M. | Incipient. | Recovered. |
| John Smith, M. | Incipient. | Recovered. |
| John French, M. | Incipient. | Recovered. |
| Hugh M'Guire, M. | Incipient. | Recovered. |
| John Daly, captain's clerk | Incipient. | Recovered. |

| NAMES. | STAGE. | RESULTS. |
|---|---|---|
| Anthony Francis, S. | Purulent. | Died. Ufed the Digit. 21 days. |
| Luke Bunter, S. | Purulent. | Died. Ufed ditto 14 days. |
| Matthew Shires, S. | Purulent. | Died. Ufed ditto 15 days. |
| Daniel Rufe, S. | Purulent. | Died. Ufed ditto 17 days. |
| Benjamin Chapman S. | Purulent. | Died. Ufed ditto 10 days. |
| James Bridon, S. | Purulent. | Died. Ufed ditto 19 days. |
| John Wilfon, S. | Purulent. | Died. Ufed ditto fix weeks. |
| —— Jamefon, S. | Purulent. | At one time much better; now dying. |
| Patrick M'Elwain, S. | Purulent. | Difcharged much better. Since died in Ireland. |
| Andrew M'Tegart, S. | Purulent. | Difcharged much relieved; but Incipient. |
| James Bradley, S. | Purulent. | Ditto, much relieved, ditto |
| James Williams, S. | Purulent. | Ditto, ditto, ditto. |
| Charles M'Carty, S. | Purulent. | Ditto, ditto, ditto. |
| Mr. Todd | Purulent. | Ditto, ditto, ditto. |
| William Thompfon, S. | Purulent. | Ditto, ditto, ditto. |
| Stephen Cornwall, S. | Purulent. | Ditto, ditto, ditto. |
| Robert Edwardfon, S. | Purulent. | Ditto, ditto, ditto. |
| John Miniman, S. | Purulent. | Ditto, ditto, ditto. |
| James Johnfon, S. | Purulent. | Ditto, ditto, ditto. |
| James Stone. | Purulent. | Ditto, ditto, ditto. |
| James Madden. | Purulent. | Ditto, ditto, ditto. |
| Benjamin Eve, S. | Purulent. | The Digit. completely failed, altho' he took it fix weeks. |
| George M'Nally. | Purulent. | Much relieved. |
| Robert Squire, S. | Incipient. | Much relieved. |
| David Rollins, S. | Incipient. | Difcharged, much relieved. |
| Daniel Gallagan, S. | Incipient. | Ditto, ditto. |
| William Cafey, S. | Incipient. | Ditto, ditto. |
| Robert Kelly, S. | Incipient. | Ditto, ditto. |
| Robert Taylor, S. | Incipient. | Ditto, ditto. |
| Edward Hofkins, M. | Incipient. | Ditto, ditto. |
| James Hutton. | Incipient. | Ditto, ditto. |
| James Candy, M. | Incipient. | Ditto, ditto. |

*** Names marked S. are Seamen. Thofe marked M. are Marines.

